# Creating pre-conditions for change in clinical practice: the influence of interactions between multiple contexts and human agency

**DOI:** 10.1108/JHOM-06-2020-0240

**Published:** 2020-10-27

**Authors:** Michelle Myall, Carl May, Alison Richardson, Sarah Bogle, Natasha Campling, Sally Dace, Susi Lund

**Affiliations:** School of Health Sciences, University of Southampton , Southampton, UK; Faculty of Public Health and Policy, London School of Hygiene and Tropical Medicine , London, UK; Clinical Academic Facility, Southampton General Hospital, University Hospital Southampton NHS Foundation Trust , Southampton, UK

**Keywords:** National Health Service, England, Implementation, Organisational change, Context, Agency, Treatment Escalation Plan, Normalisation process theory, Case study

## Abstract

**Purpose:**

The purpose of this paper is to explore what happens when changes to clinical practice are proposed and introduced in healthcare organisations. The authors use the implementation of Treatment Escalation Plans to explore the dynamics shaping the translational journey of a complex intervention from research into the everyday context of real-world healthcare settings.

**Design/methodology/approach:**

A qualitative instrumental collective case study design was used. Data were gathered using qualitative interviews ( *n* = 36) and observations ( *n* = 46) in three English acute hospital trusts. Normalisation process theory provided the theoretical lens and informed data collection and analysis.

**Findings:**

While each organisation faced the same translational problem, there was variation between settings regarding adoption and implementation. Successful change was dependent on participants' ability to manage and shape contexts and the work this involved was reliant on individual capacity to create a new, receptive context for change. Managing contexts to facilitate the move from research into clinical practice was a complex interactive and iterative process.

**Practical implications:**

The paper advocates a move away from contextual factors influencing change and adoption, to contextual patterns and processes that accommodate different elements of whole systems and the work required to manage and shape them.

**Originality/value:**

The paper addresses important and timely issues of change in healthcare, particularly for new regulatory and service-oriented processes and practices. Insights and explanations of variations in implementation are revealed which could contribute to conceptual generalisation of context and implementation.

## Introduction

This paper focusses on a long-standing problem for healthcare: that of changing clinical practice ( Davidoff and Batalden, 2005 ). Altering established behaviour of any kind is difficult. It is particularly challenging in healthcare because of the complex relationships between professionals, patients and carers and the interplay with contexts in which change is to be operationalised. This paper explores how changing clinical practice in three English acute hospital trusts, through the proposed introduction of a newly developed complex clinical decision-making intervention, a Treatment Escalation Plan (TEP), necessitated interactions between the participants attempting to make changes and multiple contexts which influenced and shaped the process within the organisation. The key question to be addressed is “what happens when changes to clinical practices are proposed and introduced in healthcare organisations”?

### What is the role of a treatment escalation plan?

In the UK TEPs have been in use since 2006 ( Mercer, 2009 ) and internationally since the 1990s ( Schmidt *et al.* , 2014 ; Thomas *et al.* , 2014 ; Godfrey *et al.* , 2012 ). They offer an alternative approach to stand alone do not attempt cardiopulmonary resuscitation (DNACPR) orders, which provide instruction in the event of a cardiorespiratory arrest, and are designed to avoid futile invasive procedures, but for which there is strong evidence of variation and sub-optimal practice ( Gibbs *et al.* , 2016 ; Hurst *et al.* , 2013 ). DNACPRs have also been the focus of ethical concern and legal challenge ( Sokol, 2016 ). In the UK such concerns led to a call to prioritise situating DNACPR decisions within a nationally recognised overall plan for emergency care and treatment ( Perkins *et al.* , 2016 ), and to the development of a national TEP process, the Recommended Summary Plan for Emergency Care and Treatment (ReSPECT) ( Fritz *et al.* , 2017 ). TEPs situate the DNACPR decision within an overall plan for treatment, offering a process for supporting clinical decision-making that take into account the treatment preferences of patients and their families, and define the scope and limits that can be applied to care ( Fritz *et al.* , 2013 , 2017 ; Mockford *et al.* , 2015 ; Jesus *et al.* , 2014 ; Schmidt *et al.* , 2014 ).

Evidence suggests that the clinical community welcome and support TEP use as a plan that is transportable between multiple clinical contexts and settings, not service or disease specific, and shared between professionals, patients and families ( Mockford *et al.* , 2015 ; Field *et al.* , 2014 ). Increasingly UK healthcare providers are expected to consider and discuss patient preferences for treatment and care and this has informed the development and dissemination of ReSPECT ( Pitcher *et al.* , 2017 ).

Discussions about a patient's wishes for treatment and care in the event of clinical deterioration have particular relevance in times of crisis, such as the global coronavirus disease 2019 (COVID-19) pandemic that first emerged in late 2019 ( Liverpool, 2020 ). In these situations TEPs can offer a framework for prompting and conducting conversations about a patient's wishes in the event of acute life threatening illness, especially for those with chronic life-limiting diseases ( RCUK, 2020 ). This not only enables patient and family preferences to be taken into account, but gives confidence to clinicians to have the discussions. TEPs can preclude a moral imperative to treat, if that is the patient's wish, reducing the burden on healthcare professionals, patients and families and they have the potential to reduce the need to “react and treat” in times when usual communications and interactions between healthcare professionals and family members are difficult.

However, implementing TEPs into routine practice can be challenging at an individual, organisational and system level, making them difficult to introduce and embed in a healthcare setting. It has been argued this can be attributable to the complex and contentious nature of TEPs ( Cummings *et al.* , 2017 ; May *et al.* , 2020 ). TEPs are an example of a contentious intervention in practice because they are distinguishable from other complex interventions in that they involve challenging interactions between patient, family and clinicians which can arise because of their moral purpose and value; and seek to formalise or make concrete clinical judgements in the face of rapidly changing situations. Contentiousness can arise at any point in the life cycle of an intervention. In this way TEPs characterise a contentious intervention which can include one or more facets of their workability, legitimacy and integration with existing practices.

### Context, agency and implementation

The role of context is well established in implementation science and in recent years attention has been paid to understanding the relationship between context and implementation in mediating change processes in healthcare organisations ( Damschroder *et al.* , 2009 ; Pfadenhauer *et al.* , 2015 , 2016 ; Dopson and Fitzgerald, 2005 ; Kaplan *et al.* , 2010 ; Aarons *et al.* , 2011 ; Bate, 2014 ; Robert and Fulop, 2014 ; Squires *et al.* , 2015 ; May *et al.* , 2016 ; Maniatopoulos *et al.* , 2020 ). This paper identifies and characterises elements of contexts that influenced the introduction (or not) of TEP and the nature of the work to manage them.

In defining context a number of existing frameworks, exploring the recursive relationship between human agency and context, are drawn upon in this paper ( Greenhalgh *et al.* , 2016 ; Damschroder *et al.* , 2009 ). Rather than a physical or environmental setting in which change takes place, or a backdrop for implementation ( Kitson *et al.* , 1998 ; McCormack *et al.* , 2002 ), this paper views context as a dynamic and fluid entity involving multiple interactions and mobilisation by actors. ( Fitzgerald *et al.* , 2002 ; Dopson and Fitzgerald, 2005 ). Context is characterised as a set of interactive processes encompassing the social, cultural and political, constantly changing and evolving over time. It is characterised by complex connections, interdependencies and interactions, not only between human actors but also between actors and context that impact on change ( Dopson *et al.* , 2008 ; Pfadenhauer *et al.* , 2017 ; Kirk *et al.* , 2016 ; May *et al.* , 2016 ). The definition used here is also informed by the conceptual and theoretical contributions to understanding implementation processes by normalisation process theory (NPT) ( May and Finch, 2009 ) which has engaged with the problem of dynamic elements of context as both the sources of resources that can be mobilised by participants in implementation processes ( May, 2013a ) and as sources of turbulence that affect their course and direction of complex systems ( May, 2013b ). Through the lens of NPT the translational process that moves healthcare interventions beyond the closed systems of research studies into the open systems of “real world” clinical practice depends on these interactions and negotiations between their participants and contexts.

In this paper a move away from contextual factors that influence implementation, to considering contextual patterns and processes that accommodate different elements of “whole systems”, and the work required by participants to manage and shape these to bring about change is explored. It shows how participants interact and mobilise aspects of context, and how individuals can influence context and shape action as they negotiate the normative and relational environment in which they are set. It is argued the relationship between participants and contexts in which change occurs is reciprocal, a two-way non-linear process which is often under-played. This may help to both understand how some interventions fail to gain traction in everyday practice settings and why it can be difficult to de-normalise already embedded practices ( Malham *et al.* , 2019 ; May *et al.* , 2016 ; Greenhalgh *et al.* , 2017 ).

Findings presented here are drawn from a wider research programme that investigated the dynamic processes involved in the implementation of a TEP within and across healthcare systems in England. The work was underpinned by the research question: *How are rules and resources implemented and integrated in practice across a whole system and how are variations in implementation and integration, experienced and explained?* This sought to understand how participants in the implementation process conceptualised the value and purpose of a TEP, and its integration into existing practices. It also looked to extricate both factors and processes that influenced successful TEP implementation in participants' organisations, and the intersection between local TEP initiatives and a national TEP process.

## Design and methods

### Theoretical framework

NPT provided the theoretical lens for the study ( May and Finch, 2009 ). NPT offers a framework to support understanding of the dynamic processes encountered during implementation and is concerned with aspects of individual and collective behaviour and the mechanisms that drive them ( Murray *et al.* , 2010 ; Finch *et al.* , 2013 ). NPT is concerned with how participants come to make sense (coherence), engage and commit (cognitive participation), enact (collective action), and reflect upon and evaluate (reflexive monitoring) a practice. It is focussed on how these elements are mobilised by participants in the implementation process ( May, 2013a ); act as sources of tumult and turmoil that influence the direction of implementation ( May, 2013b ) and how participants in implementation processes interact with other actors and elements in the context in which they are set ( May *et al.* , 2016 ). NPT has been shown as a valuable heuristic device for increasing understanding of implementation journeys of a variety of interventions, including TEPs, in healthcare settings ( McEvoy *et al.* , 2014 ; Cummings *et al.* , 2017 ; Huddlestone *et al.* , 2020 ). In this study, NPT provided a useful set of conceptual tools to understand the process of translating TEP into practice, informing the development of our research question, and shaping data collection and analysis.

### Study design

We employed a qualitative instrumental collective case study design in which each participating healthcare organisation was conceptualised as a “case” ( Stake, 1995 ). The unit of analysis was the process to introduce TEP in order to explicate the relationship between participants and contexts. Each case offers a narrative of the implementation journey in each organisation, constructed from empirical findings generated from interviews and observations. Approval for the study was obtained from the University of Southampton Research Ethics Committee (Ref: 24,886).

### Setting and participants

Research was carried out in three National Health Service (NHS) acute hospital trusts in one region in England. Despite attempts to recruit primary care organisations to the study none chose to participate and we acknowledge this as a potential limitation. Purposive sampling was used to identify participants who represented different stakeholder groups and roles in settings considering implementing a local TEP or the national ReSPECT process ( Table 1 ). These included healthcare professionals, with knowledge and experience of clinical situations where TEPs would be operationalised, and members of implementation working groups established within and across trusts to undertake different types of work required prior to implementation.

### Data collection

#### Interviews

Interviews ( *n* = 36) were conducted between January 2017 and July 2018 by MM, SL and NC and audio-recorded (with consent). Average duration was between 25 and 60 min. Interviews were carried out using a topic guide informed by NPT ( May and Finch, 2009 ) and sought to capture data on the value and purpose of TEP (coherence), participants involved (cognitive participation), the work involved (collective action) and appraisal undertaken as part of the process to introduce and implement TEP (reflexive monitoring).

#### Observations

Observations of locality-wide workshops, held to explicate views on the scope, design, content and process of TEP, and TEP/ReSPECT implementation working groups, were undertaken by MM, SL, SB and AR. Over 60 h of observation were carried out over the course of the study resulting in 46 sets of notes for analysis. Observations were crucial to providing insight into aspects of the TEP implementation journey that could not always be captured by interviews. They enabled a real time understanding of the different contexts intersecting in the process to introduce a TEP and an opportunity to witness first-hand the interactions between different participants and contexts to be managed and navigated.

### Data analysis

A sample of interview transcripts and fieldnotes were read and coded independently by MM, SL and NC using initial codes, developed from *a priori* concepts derived from the study research questions against the backdrop of NPT, and new codes derived from the data. Data clinics were held to review and refine codes to determine fit with the initial code list and validity of codes. Once a set of codes were agreed a framework was developed and applied to the whole data set. Interviews were transcribed verbatim and transcripts, along with observation fieldnotes imported into NVivo Version 10 (QSR International Pty Ltd) software to aid coding and general management of the data. Codes were collated into potential themes that were discussed at subsequent data clinics where we also looked for patterns and connections across the themes to produce an interim analysis within and across the settings. Our analysis of the relationship between context and agency in trying to initiate change developed out of a series of team discussions where we generated key ideas about different contexts that required navigating and the work involved to manage them. We also used matrices and charting to facilitate comparisons which identified differences between settings ( Ritchie and Lewis, 2003 ) as well as ongoing discussions within the research team to test our interpretations.

## Findings

To explicate the relationship between participants and context, and their influence on each other, we offer three case studies of the journey to introduce a change to practice in each organisation. The starting point of which was an initial expression of interest in designing and co-producing a local TEP. However, the introduction of ReSPECT offered a turning point as the decision was made to continue with considering implementation of a local version or move towards the national ReSPECT process.

### Case 1: site A

At the outset, in terms of the goals of care in place, the trust was utilising an established DNACPR order, for use by the whole of the local healthcare economy. This had been implemented by the trust six years previously and taken time to embed, leading to concerns by some about its integration with or replacement by a TEP.

Certainly here in [name of Trust], we have a local DNACPR which is very well established and fairly well done system of recording if somebody is not for attempted resuscitation. The danger for me then is that something that is working well potentially is not cast aside and replaced with something else. So my worry would be losing what is already working well. [Senior Nurse ID G11]

In its electronic format, the DNACPR was considered to offer a well-functioning system but it was acknowledged that it incorporated one specific treatment decision and only initiated conversations with patients who were seriously unwell.

Initial discussions regarding TEP implementation coincided with an upcoming review of the trust resuscitation policy and development of other goals of care plans, and presented an opportunity to make a change. A working group was convened to consider the trust's position in relation to implementation. A chair was appointed and the core membership comprised a range of clinical specialities. Regular meetings were held over a two-year period to consider, undertake and delegate the work needed for implementation. Initially, there was strong support from clinicians who could see value in aligning the introduction of TEP with the trust's strategic priorities, including reduction in patient complaints and admission avoidance. While improving patient experience was acknowledged, the focus was on meeting targets. Work began to make the case for change and discussions focussed on the target patient population, education and training required, and bringing about a culture change within the organisation. Widening the working group membership was considered important to obtaining buy-in and while engagement routes and potential individuals were identified, these rarely materialised.

In reviewing the actions of the previous meeting, work had been done to invite more people to join the group. This included a clinician from Emergency Care. There was also discussion about including other specialisms for which it was agreed “it was worth exploring”. [Observation Field notes, Site A, 8th Working Group Meeting]

Low attendance at today's meeting and new members present at the previous couple of meetings were absent. It was reported that the Emergency Care Clinician who had been invited to join was now unlikely to participate and they would need to identify someone else. An Emergency Medicine Consultant was suggested as a potential but no action was assigned for someone to contact them. [Observation Field notes, Site A, 11th Working Group Meeting]

From the outset, fundamental issues evident within the group early on remained unresolved. In particular, a preference for a local TEP rather than the ReSPECT process, despite the latter having organisational support and recognition that ReSPECT was being promoted for national adoption. Previous acknowledgement of gaps in the DNACPR, with regard to treatment escalation, were superseded with increasing concerns that TEP would replace the DNACPR, as the national process required incorporating DNACPR within ReSPECT. In essence a TEP was sought that was locally contextualised, leading to a reluctance to relinquish the DNACPR that persisted.

In addition, dissatisfaction with the content and format of the ReSPECT form led to a pre-occupation with the form, rather than the overall process. It also revealed some concerns about risk and uncertainty with regard to the national process that extended to professional fears regarding clinicians' ability to carry out the work of ReSPECT, which necessitated conducting sensitive conversations with patients and families.

Observation fieldnotes recorded on several occasions that the issue of “ *staff being scared to have the conversation due to lack of confidence”* and *“a need to increase confidence in having the conversation across the board”* being raised at working group meetings.

There was a lack of coherence by some working group members of the difference between TEP and other end of life care plans, leading to a lack of concordance regarding the purpose and value of TEP. Agreement also failed to be reached on the target population for TEP and if it should be initiated in the community or acute sector. Similarly, a lack of consensus persisted with particular implementation issues such as resources, ownership and leadership.

After the first six months, there was a gradual loss of momentum and commitment appeared to wane. Meetings were poorly attended, with only two or three members present at some, the chair was often absent resulting in actions from previous meetings not being followed up, and work between meetings was limited or did not happen. As progress slowed, gradually resistance grew from some who had originally been supportive and a professional defensiveness about TEP implementation became increasingly evident. By the end of the second year, no plans were made to hold further meetings and attempts to introduce TEP ceased.

### Case 2: site B

The trust had a history of implementing change, with a preference for national rather than local solutions. They had been early adopters of previous pathways for delivering care to patients at end of life and were experienced at enacting both local and national goals of care programmes. The trust used the national DNACPR form for all *do not resuscitate* decisions, having not signed up to the local version in operation across the neighbouring region, following concerns from the trust's legal team regarding accepting a DNACPR form that went to different care settings. The use of a non-transferrable DNACPR that was only suitable for use in the hospital resulted in limited cross boundary communication. Frustrations were also expressed regarding the trust requirement of a new form having to be completed each time a patient was admitted, even if readmission was within a few hours of discharge, leading to clinicians feeling disempowered to change inefficient processes.

The hospice linked to the trust used a local TEP at discharge and there was a history of external pressure for the hospital to do the same. While there was support for a TEP process in the trust there were concerns that this local TEP, developed for use in the community, was not fit for purpose in a hospital setting. This led to any attempts to impose the TEP being rejected in favour of waiting for a national solution. The lack of an effective DNACPR and TEP process suggested there was potential to make a case for change.

Initial interest in working towards TEP implementation led to the setting up of a working group with a range of specialities represented, and potential individuals identified who could extend the membership. At the time, a national TEP process was not on the immediate horizon but the group decided to go ahead. At the inaugural meeting, there was strong engagement. Agreement was reached about the target population for TEP, and consensus that the process was not just about the form but more importantly the conversation with patients and families. This was an opportunity to bring about a change in culture within the organisation and improve patient care and experience and the group were clear as to what mattered most.

The discussion moved on to what was important with regard to TEP. It was agreed it was less about the form and there should be a focus on cultures and behaviours to understand why people do not do things. Keeping the form simple and transferrable between settings was key. The patient should be the custodian of the form with an electronic version so it could be shared. A clear way to indicate when decisions had been superseded was crucial and the form needed to be workable for confidential use by junior doctors and nurses. [Observation Fieldnotes, Site B, 1st Working Group Meeting]

However, while there was initial enthusiasm, little was discussed in terms of next steps or an action plan. Eight months elapsed between the first and second meetings. By the time of the second (and final) meeting, plans were underway to introduce the national ReSPECT process. However, organisational priorities had now changed with an imminent inspection from the independent health and social care regulator. The chair reported challenges around securing time commitments from the group and a lack of buy-in at senior level.

It feels that nobody has got any time for it and it's taking a back burner. For me, the first thing is that everybody actually wants to do it particularly from the kind of management perspective … if you don't have everyone engaged and knowing the reason why it's needing to be done, then you fail at the first hurdle. So, I think, it's--- it's having that support from management preferably with some form of resource and then having the shop floor support. [Consultant, Site B, ID SO4]

The interest and engagement that was evident early on had largely evaporated. While a financial incentive was mooted as a way of renewing interest, there was a reluctance to pursue implementing the national process and go it alone if other trusts in the locality did not adopt. The Working Group Chair continued to engage in regional meetings with other organisations who were considering adopting ReSPECT but no further meetings to consider its introduction in the trust were convened.

### Case 3: site C

The use of care pathways was well established within the trust, with regular audits of their completion, and education and training initiatives to support their use. The locality wide DNACPR was in operation and viewed as functioning well. However, it was recognised that there was a gap when it came to conversations about goals of care that were not focussed solely on resuscitation. A need to improve treatment decision-making and work to make the case for change, including an audit of “out of hours” calls, was already underway when several key protagonists were alerted to the opportunity in the region to develop a local TEP. From the outset, coherence around the purpose of a TEP was generated and there was a clear collective vision within the working group as to its value. The drive to implement focussed firmly on the benefits for patients and their families.

I think, there's huge value for all professionals involved to make sure that we're meeting the patient's expressed wishes about their medical treatment and, importantly, I think for out of hours care where there's a lot of uncertainty, a lot of limited professionals around who may or may not know the patient. So, if a patient particularly deteriorates out of hours then everybody, including the nursing staff, know how they are to treat and manage the care for that person, again, in line with the person's preferred wish and with family involvement too. Whether that's including a DNACPR or whether it's just escalation to a high dependency unit, or not, so that we're not doing unnecessary investigations or interventions [Nurse, Site C, ID B07]

There was evidence to suggest that TEP was viewed as a means to offer person-centred individualised care with recognition that the conversation was more important than the form.

TEP actually prompts patients to think about this and to discuss what their wishes are with their close family, and their carers, and their medical team, so that we can then make the right decisions at the crisis point, or have more information about what the patient's wishes are because often we don't actually have all of that information. [Consultant, Site C, ID B05]

While there was some initial uncertainty about the target patient population, this was quickly resolved and the process to implement commenced. A working group, convened by a palliative care consultant, comprised membership from a range of clinicians. However, while there was a determination for TEP to be seen as broader than a palliative care initiative, attempts to shift the leadership to other specialities were unsuccessful and with no one else to drive it, the role of chair remained with palliative care for the duration of the process.

The requirements of the leadership role changed over the implementation process from promoting the value of TEP, to how they were going to make it happen and engaging others outside of the organisation. While the role of the leader continued to be important, this lessened as the collective influence and strength of the group increased. A keenness to work cross-organisationally and involve others outside of the trust was voiced from the start and in-between meetings new members were engaged from the community, general practice, ambulance service, and local healthcare planning and commissioning services.

Recognition of the impact of implementing a TEP on partner agencies within the local health and care communities led to key stakeholders being informed and included early in the process. This was essential to achieving buy-in and linking with other networks ensured that the group did not work in isolation and had legitimacy to make decisions. In addition, the diversity of the membership and continual engagement with a range of stakeholders served to strengthen the group and provided them with the credibility to challenge dissenters who sought to block or derail implementation. At several points in the implementation journey significant opposition, or at a minimum neutrality, was expressed by individuals within the trust and the wider clinical community. This necessitated the management of complex relationships and interactions by the group whose approach was always to listen to and engage those opposed to implementation.

Patient representation was also considered integral to the membership and the patient voice was important to maintaining group momentum when clinicians were sometimes in danger of losing their way and becoming enmeshed in the process and the challenges of reaching consensus and agreeing implementation plans. This was evident from the comments of one patient contributor who urged the healthcare professionals “I *don't care what colour the form is, or what system [is used] please find a way to make it work* ”. [Observation Fieldnotes, Site C Working Group 8]

The working group functioned as a collaborative with each member having their own area of responsibility and drawing on their specialist knowledge, derived from both professional and personal experience. From the first meeting there was recognition that processes were required to manage the introduction of TEP in the trust. Terms of reference and a reporting structure were developed and by the third meeting, these were agreed and then reviewed during key points in the process, for example following the transition from a working group to a ReSPECT implementation network when the decision was made to pursue the national process. A core working group and identified leads for various aspects of the implementation work meant consistent progress was made on completing different tasks. This included development and application of a formalised implementation framework; a sustained communication strategy involving both practitioner and non-practitioner routes; training and education sessions to engage staff members and raise awareness; and strategies to overcome technology and practical resource issues. Engaging with the public to avoid misinformation about ReSPECT, and its purpose, and prevent negative media attention was also seen as important work of the group.

The Chair raised concerns about possible media interest that could be either very positive or extremely negative. They said there had already been a fairly negative piece in an English tabloid newspaper with regard to ReSPECT so it was necessary to consider early engagement with local media as they start moving on this. [Observation Fieldnotes, Site C, 3rd Working Group Meeting]

Following sustained levels of engagement and commitment and a process characterised by a pragmatic and “solutions not problems” approach to implementation, ReSPECT was introduced in the trust two years after the first meeting was held.

## Discussion

The focus of this paper has been to explicate the relationship between agency and context when attempting to bring about change in an organisation. The use of case study design enabled a rich and detailed insight to be developed within and across multiple organisational settings. This helped to identify and characterise the internal and external dynamics of implementation processes and understand how interactions between participants and contexts can influence change. Using NPT enabled the identification of the different types of work needed to manage contexts during the translation phase. Findings show that while each organisation faced the same implementation problem, there was variation between settings with regard to adoption. We suggest this can be explained by participants' interaction with, and mobilisation of, aspects of contexts and the work carried out to shape and manage contexts.

We identified a number of contexts that operated at multiple levels (micro, meso and macro) which helped to explain and understand organisational change and the reasons for variation across the different settings ( Maniatopoulos *et al.* , 2015 ; Hunter *et al.* , 2015 ). Previous studies have shown that implementing complex and contentious interventions presents challenges at the individual, organisational and systems level. It is these interaction level processes that make an intervention contentious, as well as making it challenging to introduce and embed ( Cumming *et al.* , 2017 ). In this study there was not always a clear demarcation between these three levels of context and the lines between were often blurred. On their own each context did not exert influence on whether change happened. Instead there were different intersecting contexts with complex connections, interdependencies and interactions played out over multiple timeframes within and between organisational settings ( Maniatopoulos *et al.* , 2020 ). In the discussion that follows, we identify and characterise the contexts and the work required to create a receptive context for change.

### Political and regulatory context

All healthcare organisations are influenced by the political and regulatory context in which they are situated and by broader social processes in which actions take place ( Shaw *et al.* , 2017 ). For the organisations in this study, one aspect of the political and regulatory context was the documentation of decision-making around future treatment and care. The legacy of legal issues in the UK with regard to sub-optimal discussions around DNACPR decisions made by clinicians ( Sokol, 2016 ) created an ethical and legal imperative of involving patient and family members in DNACPR decisions and led to a professional recognition of a need for change that created the political pre-conditions for ReSPECT. However, while the use of ReSPECT is advocated by UK professional bodies, including the Royal College of Physicians ( Bailey and Cogle, 2018 ), it operates within a de-centralised health service, resulting in a lack of a national mandate and no regulatory imperative for its implementation. Organisational responsiveness to national policy and guidance has been shown to promote value amongst staff members and thereby influence implementation of complex healthcare interventions ( Fritz *et al.* , 2013 ; Cummings *et al.* , 2017 ). In the organisations included in this study the absence of a mandate of ReSPECT influenced the direction of travel for its implementation and required work to generate coherence and cognitive participation, around not only the purpose and value of introducing a TEP, which would replace existing practices, but also as to the merits of implementing ReSPECT or a local version.

In order for coherence to be achieved, those driving change had to believe, and demonstrate to others, how a local TEP or ReSPECT would offer something better for patients, families, clinicians and the organisation, particularly in an environment where there was no strong imperative to make that change. For this reason, the people leading the process required the ability and capacity to mobilise others and encourage them to be participants, not by-standers in implementation. Here the work of active leadership was to enact, not simply react to, the context in which implementation took place ( Weick, 1979 ) and to enable collective action to shape and manage contexts to bring about change. Leadership was critical to securing cognitive participation and buy-in to the process from different levels within the organisation, including senior management. While high-level endorsement has been recognised as important to successful implementation of complex interventions ( Cummings *et al.* , 2017 ) we observed that this did not necessarily guarantee success, and the influence of determined clinicians could outweigh executive direction. In organisations where leaders could not achieve coherence, and cognitive participation was limited, implementation did not happen.

### Historical/temporal context

In all three organisations participants had to manage and navigate a historical/temporal context which operated at the local level and characterised by the “shadow of past periods and historic decisions” ( Dopson *et al.* , 2008 ) they had inherited. A legacy of previous experiences of implementation resided in the collective memory of participants. A socially constructed shared interpretation of past attempts at introducing change meant that different types of work were needed to create the right conditions at the outset of the implementation journey that would enable the constraints of the historical context to be minimised. In NPT terms, this required establishing coherence to enable participants to exploit past learning for future change and engage in reflexive monitoring to evaluate the success, or lack of, past implementation efforts ( Bevan *et al.* , 2007 ). Work was also needed to raise consciousness regarding what a TEP, local or national, could achieve for patients and families. This required creating understanding of the historical/temporal context as an opportunity, rather than a threat and reason not to introduce a change to practice. This was achieved in Site C who drew on a history of adopting initiatives to support practice, which contributed to securing cognitive participation and building a network to support implementation. In contrast, Site B was unable to generate the coherence and acquire cognitive participation in the form of a critical mass, and escape the perceived constraints of its legal legacy and relationship with other organisations in the healthcare system.

### Organisational context

One element of organisational context that was observed was willingness to commit to changing practice, which was particularly evident in Sites A & B. In Site A, this related to ReSPECT as potentially disrupting or destabilising to existing policies and procedures, specifically the local DNACPR. While this was a misunderstanding of the difference between ReSPECT and DNACPR processes, the result was a reduced impetus to change. ReSPECT could not compete in an organisation overwhelmed by normalised practices and insufficient levels of coherence and cognitive participation needed to achieve the collective action required to make a case for change or produce the evidence to do so. Previous studies have shown that one facet of successful implementation lies in minimal disruption to existing working practices and its differentiation from other practices to be valued ( May, 2013b ). Furthermore, clarity regarding the practices to be discontinued, preventing unnecessary duplication and clear identification of the benefits of the new intervention have also been identified as key to adoption ( Cummings *et al.* , 2017 ). In Site B, an apparent cultural stubbornness and a tendency to be risk adverse manifested in a preference for a national solution but reluctance to go it alone with implementing ReSPECT. Instead, they preferred to wait for sufficient system-wide engagement, signifying a need to be driven rather than to drive the process. This suggests a reactive strategy representing facets of existing organisational culture, rather than clinical priorities, and an environment where clinicians did not feel empowered to act or initiate the work needed to lead change.

The extent to which organisations were inwardly or outwardly focussed also contributed to the implementation effort. The willingness and/or ability to engage beyond one's own organisation and establish networks with the wider health and care community was important for ensuring the translation of ideas within and across organisations. To be successful, work had to be invested in building and sustaining a group or network who were committed to the long haul, where there was engaged participation, willingness for sustained collaboration, sharing of power at an individual and organisation level, and clear objectives and a process to achieve them ( Goodman *et al.* , 2017 ). It was not simply a matter of securing cognitive participation, but ensuring the enrolment of the “right” people, inside and outside of the organisation, to carry out the collective action required for the work of implementing change. Furthermore, generating coherence around the purpose of the network was vital to members understanding their individual and collective roles within the group.

In this study, Site A could be characterised as an inwardly focussed organisation. Attempts to make sense of the purpose and value of TEP, and particularly ReSPECT, were confined within their own boundaries rather than the whole system and a focus on the operationalisation of ReSPECT within specific clinical specialities rather than system-wide. Analysis of Site B pointed towards an environment that could be regarded as inflexible and impermeable. Its negative historic relationship with the adjacent healthcare system, led to a resistance to engage with other trusts. Despite individual level commitment from a small number of working-group members, the work needed to engage and mobilise within, l *et al* one beyond, their organisation did not materialise. Site C, on the other hand, sought to engage and enrol members who were internal and external to the organisation from the outset. Participants were drawn from a range of professional groups, patient and public representatives and throughout the process they sustained and maintained intergroup relationships. Again, this appeared to be attributable to the work of active leadership mediated through a local leader, regarded as credible by members, and the presence of existing sound relations and informal networks, key to enabling collective action from a range of participants. Collective action, not only comprised tangible activities such as enacting communication and education/training strategies, resolving resource issues and having a clear process, but those less palpable. For example, establishing trust and confidence in the network and others in the change process, and managing those who sought to derail the implementation effort. Furthermore, in Site C reflexive monitoring through an established process of audit and evaluation contributed to generating coherence, obtaining cognitive participation and shaping collective action.

### Interactional contextualisation and the moral dimension of practice

The professional context was a complex mediator of change and consequently more challenging to manage. It was in this context that contentiousness in practice was encountered encompassing aspects of individual professional roles and identity, professional fears and concerns, and the moral dimension of practice. In all sites, improving the quality of the patient and family experience by involving them in decision-making about future treatment and care was not disputed by participants as the “right thing to do”. This reflected the moral dimension of practice and the shared moral order ( Harré and Van Langenhove, 1999 ) governing healthcare with regard to fulfilling a moral obligation as a healthcare professional and pursuing a correct course of action.

Professional concerns related to how introducing TEP/ReSPECT could change the nature and expectations of the clinicians' role, legitimacy of participation and willingness to undertake collective action. This manifested through an apprehension revealing professional fears and articulated through a discourse of risk and risk mitigation ( Gale *et al.* , 2016 ), related to the initiation and enactment of TEP/ReSPECT, the requirement for decisions to be documented, and for a clinician to place trust in and operationalise decisions by others or multiple others. In this way, the operationalisation of TEP/ReSPECT involved a network of actors and decisions that were distributed across organisational space, time and professional groups ( Rapley, 2008 ) requiring changes to the existing professional script and threat of exposing oneself to sensitive conversations. This was further intensified through a lack of elasticity and malleability in the ReSPECT process, which prohibited local interpretation or flexibility regarding use, and concerns about the ability of those tasked with its operationalisation. In NPT terms, the role of relational integration (the knowledge work undertaken to build accountability and maintain confidence) and skill set workability (the appropriate allocation of work to operationalise practice) were pre-conditions for collective action in the process to introduce change as they reflected participants understanding of the work that would be required to manage an uncertain trajectory and associated implications.

This context operated at the micro level and its management and navigation were dependent on individual capacity, motivations and perceptions, perhaps more so than in other contexts identified. Here, perception of context and participants' anticipation of how TEP/ReSPECT might alter professional roles was used to justify their actions and behaviours. Participants drew on a combination of cognitive and emotional judgements to make sense of this context that they then enacted, and in turn, affected their ability or willingness to bring about change ( Weick, 1995 ). This was particularly evident where professional defensiveness about TEP/ReSPECT and perceptions of challenges to individual authority were observed. Furthermore, through their actions, participants opened up the possibility of resisting a shared moral order and instead creating a personal moral order ( Van Langenhove, 2017 ) based on individual motivations and influenced commitment and engagement in collective action to enact change.

## Conclusion

Through a focus on attempts to introduce a TEP into healthcare organisations, this paper offers empirical evidence to demonstrate how change in clinical practice is influenced by interactions between participants and multiple contexts, and how successful change is dependent on the extent to which participants have the ability to manage and shape these contexts. Interaction between participants and context can be influenced by the intervention and this is particularly evident when the intervention is contentious in nature. Applying the lens of NPT enabled an understanding of the work needed to negotiate and navigate these contexts in the implementation process and revealed how the work itself was reliant on individual capacity to create a new, receptive context for change. The active role contexts play in fashioning pre-conditions for change means that no context is discreet. For this reason, managing them is a complex, interactive and iterative process, whereby participants influence the flow of events and context is constructed and re-structured to liberate or mobilise change. A recommendation of this paper is, therefore, that an assessment of the understanding of organisational and relational requirements needs to be taken into account and acted upon prior to implementation. Undertaking such a pre-implementation assessment, appraising individual, team and organisations of levels of preparedness before embarking on implementation will identify barriers and facilitators to successful implementation, and recognise what is already in place to support participants involved in this challenging work.

## Figures and Tables

**Table 1:** Key informants

Key informants	NHS Trust 1	NHS Trust 2	NHS Trust 3	Primary care and hospice	Ambulance trust	Other	Total
Medical Consultants	7	2	3	2			14
Junior Doctors	2		3				5
Senior Nurses	4	1	4				9
General Practitioners				2			2
Paramedics					2		2
NHS England						1	1
ReSPECT National Working Group						3	3
Total							36
